# Factors associated with eye examination within the past year among older home care clients: A cross-sectional register study

**DOI:** 10.1097/OPX.0000000000002300

**Published:** 2025-09-29

**Authors:** Tiina Pesonen, Johanna Edgren, Satu Elo, Aura Falck, Heidi Siira

**Affiliations:** 1 Research Unit of Health Sciences and Technology, University of Oulu, Oulu, Finland; 2 Department of Healthcare and Social Welfare, Finnish Institute for Health and Welfare, Helsinki, Finland; 3 Oulu University of Applied Sciences, Oulu, Finland; 4 Department of Ophthalmology, Medical Research Center, Research Unit of Clinical Medicine, University of Oulu, Oulu, Finland; 5 Oulu University Hospital, Oulu, Finland

## Abstract

**SIGNIFICANCE:**

International guidelines recommend that older adults undergo eye examinations every 1 to 2 years. Eye examinations can identify vision issues at an early stage, enabling timely intervention to prevent or manage impaired vision.

**PURPOSE:**

We explored the prevalence of older home care clients who had undergone an eye examination within the past year and the associated factors.

**METHODS:**

We used a cross-sectional register study design. The data were derived from the National Resident Assessment Instrument database, authorized by the Finnish Institute of Health and Welfare. We utilized data from home care clients who were aged 65 or older and had an assessment with the Resident Assessment Instrument conducted between April 1, 2022, and September 30, 2022 (n = 19,150). We used multivariate binary logistic regression to analyze factors associated with having an eye examination within the past year among home care clients.

**RESULTS:**

Twenty-six percent of Finnish home care clients had undergone an eye examination within the past year. Having an eye examination within the past year was significantly associated with female gender (odds ratio [OR] 1.12; 95% confidence interval [CI] 1.04–1.20), impaired vision, especially moderate or severe impairment (OR 3.01; 95% CI 2.70–3.36), and mild hearing impairment (OR 1.13; 95% CI 1.04–1.22). Limited physical function, both needing supervision or limited assistance in activities of daily living (OR 0.78; 95% CI 0.72–0.85) and needing extensive assistance (OR 0.60; 95% CI 0.53–0.67), and mild cognitive impairment (OR 0.86; 95% CI 0.80–0.92), as well as moderate or severe cognitive impairment (OR 0.66; 95% CI 0.59–0.74), were associated with lower odds of having an eye examination within the past year.

**CONCLUSIONS:**

A significant share of home care clients may not seek or may be unable to attend eye examinations at the recommended intervals due to various barriers, such as limited physical function and cognitive decline. The opportunity to participate in eye examinations may require targeted support services, such as assistance or transport services, or the development of innovative home-based vision care services. In addition, home care professionals’ awareness and competence regarding eye examination recommendations, available eye care services, and overall vision health should be strengthened.

Vision plays a significant role in performing everyday activities, and it is well known that impaired vision has multiple negative consequences, including decreased physical, psychological, cognitive, and social functioning.^1,2^ The risk for vision impairment increases with age, as many vision-threatening eye diseases become more common with age.^1,3^ In addition, impaired vision may result from uncorrected or undercorrected refractive errors, which can be easily addressed by updating eyeglass or contact lens prescriptions.

Older adults (≥65 years) are recommended to undergo eye examinations every 1 to 2 years, even when asymptomatic, and more frequently if necessary.^4^ In Finland, international guidelines on eye examination intervals are generally followed. Eye examinations are essential for preventing vision impairment, as they can detect eye problems early, allowing for timely treatment and updated prescriptions for corrective lenses. Furthermore, eye examinations can reveal the need for low vision rehabilitation when vision cannot be fully corrected with glasses. Therefore, impaired vision can be at least partly prevented or managed through regular eye examinations, and individual burden and suffering can be eased when visual impairment already exists. In addition, recent evidence has shown that untreated vision loss is a risk factor for dementia.^5^ A previous study found that 32% of asymptomatic patients aged 65 years or older had new critical eye diagnoses during routine eye examinations.^6^

An eye health care professional can perform an eye examination, such as an optometrist or ophthalmologist, at a health care center or optical store. The examination includes several vision measurements, such as visual acuity, refraction, binocularity, and intraocular pressure assessment. Additionally, it involves an assessment of ocular health, including an anterior and posterior examination, with an evaluation of the cornea, iris, vitreous, and retina.^7^

In Finland, the provision of eye health care is divided between the public and private sectors. Public eye health services in Finland are limited to the treatment of eye diseases and do not include regular eye examinations for asymptomatic patients. Public eye health care services are provided only in specialized health care. The private eye care sector offers eye examinations by optometrists and ophthalmologists, including prescriptions for eyeglasses, which are considered out-of-pocket expenses for patients. Many ophthalmologists have a dual role working in both sectors. In the private sector, they usually work as independent service providers.^8^

Finland has a higher optometrist-to-population ratio (2.87 per 10,000 people) compared with many other developed countries, where the ratio is approximately 1 per 10,000.^9^ Despite the number of optometrists and ophthalmologists providing eye health care services, access to a vision examination and further eye examinations may not be as straightforward as ideal. Eye health care is partly provided in the private sector, as vision correction (e.g., glasses) and screening for eye diseases are available, along with some limited treatment. At the point when an eye disease is detected or suspected, the client becomes a patient, and only a medical doctor can refer the subject to the public sector. The majority of the diagnostics, treatment of eye diseases, and eye surgeries are carried out in public eye hospitals. The private sector is accessible to anyone without a referral, but services must be paid for out of pocket. In contrast, access to the public sectors requires a referral and the presence or suspicion of a disease, with costs then partially covered by social security. The need to enhance cooperation between the private and public eye health care has been acknowledged for quite some time.^8^ Although it has become somewhat easier since the introduction of a national patient journal 1 to 2 years ago, further improvements are still needed.

Current information about the prevalence of older adults who have undergone eye examinations in Finland in the past year is unavailable. According to a previous study, 37% of older adults in high-income countries have had their last eye examination within the past 12 months.^10^ Another study from Norway suggests that 39% of adults aged 65 or older had undergone an eye examination in the past year.^11^ Based on previous research, limited physical function,^12,13^ and greater distance from residence to an ophthalmologist can limit older adults’ access to eye examinations, creating a barrier to following the recommended frequency for eye examinations among older adults.^13^ This challenge may be particularly emphasized for individuals living alone, which is often the case among older home care (HC) clients in Finland, where approximately 80% live alone.^14^ Also, a minority of older adults are likely to be aware of the recommended frequency for eye examinations. Furthermore, in the study by Vela et al. (2012), several other factors, such as older age, female gender, higher educational level, greater wealth, worse self-reported health, and wearing glasses or contact lenses, were associated with having an eye examination within a year.

In Finland, optometrists cannot independently prescribe spectacles to a person who has previously undergone an operation involving the eyeball, such as cataract surgery, or has a suspected or diagnosed eye disease, according to Article 16 of the Law of Health Care Professionals Decree 564/1994.^15^ Therefore, in most cases, older adults need to see an ophthalmologist for an eye examination when they need a new eyeglass prescription, unless they have an individual permission from an ophthalmologist allowing the optometrist to prescribe glasses. This can create a financial barrier for low-income individuals because an ophthalmologist’s eye examination is considerably more expensive than that of an optometrist. The situation may improve in the future, as the repeal of Article 16 was taken under consideration on January 1, 2025, by the Ministry of Social Affairs and Health.

Limited cognitive function is also associated with reduced participation in eye examinations at the recommended intervals.^13^ A previous study indicates that individuals with memory problems have more unmet care needs than others of similar age.^16^ Memory problems may also be a potential risk for unequal eye health care, as it is known that fewer cataract surgeries are performed on patients with memory problems compared with others of similar age.^17^ Although there is evidence that cataract surgery may improve cognitive impairment.^18^ Furthermore, a bidirectional association has been found between vision and cognitive impairment.^19^ Therefore, eye examinations are important, especially for older adults with cognitive impairment. In 2023, 54% of Finnish HC clients experienced cognitive impairment.^20^ By correcting vision, it is possible to potentially delay the deterioration of cognition and even the onset of symptoms of a memory disease.^5^ In addition, hearing impairment has been identified as an independent risk factor for dementia,^5^ and previous studies have shown an association between vision and hearing impairment.^21,22^ Therefore, it is interesting and highly necessary to investigate whether impaired hearing is associated with eye examinations at the recommended intervals.

Vision impairment carries a considerable economic burden,^23^ affecting not only individuals themselves but also their close ones, caregivers, and society.^24^ For example, vision impairment is associated with the increased use of HC services^25^ and a higher risk of falls. Since vision has been determined as one of the key elements for healthy aging,^26^ ensuring eye examinations at the recommended intervals may indirectly help maintain the physical, psychosocial, and cognitive functioning and overall well-being, and reduce fall risks for older HC clients. However, there is limited data on how many HC clients undergo eye examinations annually, as well as on the factors associated with their participation. This information is important, as HC clients often experience physical limitations and cognitive decline, which have been previously identified as barriers to attending eye examinations. Furthermore, the majority of Finnish HC clients live alone,^14^ which can further hinder their ability to follow recommended eye examination schedules, especially for those with limited physical function and cognitive decline. Therefore, investigating the prevalence of older HC clients who have undergone eye examinations within the past year and identifying the associated factors is essential, as adequate vision may promote active and healthy aging. The results of this study are important both societally and scientifically from international and national perspectives.

## METHODS

### Design and participants

We employed a cross-sectional study design. The data in this register study has been formed from the National Resident Assessment Instrument (RAI) database, which is authorized by the Finnish Institute of Health and Welfare. The RAI database contains information, for instance, on functioning, health, and the need for care of persons receiving care services for older people. RAI data are collected using standardized instruments developed by an international organization called interRAI.^27^ Two RAIs have been designed for and are used in HC: Minimum Data Set-HC and interRAI HC. In this study, we used data gathered with the interRAI HC instrument,^28^ as it contains a question about a previous eye examination.

The data used in this study were constructed in 2023, when the most recent available information was from RAI assessments conducted in 2022, during which the use of the RAI was still voluntary but already widely adopted in Finland. However, the use of the RAI has been mandatory for all Finnish HC settings and other long-term care service providers since April 1, 2023, Act on Supporting the Functional Capacity of the Older Population and on Social and Health Services for Older Persons 980/2012, 15 a §.^29^ The RAI assessments, performed by HC workers, are transferred to the Finnish Institute for Health and Welfare’s secured database twice a year (in spring and autumn). The inclusion criteria for the interRAI HC assessments in this study were: (1) at least 65 years of age and (2) assessments performed between April 1 and September 30, 2022 (one assessment period). The study sample was 19,150 HC clients.

### Variables

#### Physical function

Physical function was measured using the activities of daily living (ADL) hierarchy scale (0 to 6), which measures an individual’s degree of dependence on four activities: personal hygiene, toilet use, locomotion, and eating.^30^ In this study, the scale was used as a categorical variable, where scores 0 indicate independence, scores 1 to 2 indicate the need for supervision or limited assistance, and scores ≥3 suggest a higher level of dependence.

#### Cognition

Cognition was measured using the cognitive performance scale.^31^ In this study, the scale was used as a categorical variable, where scores 0 to 1 indicate normal cognition, score 2 suggests mild impairment, and scores 3 to 6 indicate moderate to severe impairment.

#### Vision

Vision was determined using the vision classification (0 to 4) from the RAI assessment, where a higher score indicates more severe impairment.^28^ In this study, vision was categorized into three different categories, where 0 indicates normal vision (ability to read normal text on a newspaper or book), 1 suggests mild impairment (problems reading normal text), and scores 2 to 4 indicate moderate to severe impairment (inability to see large print, such as newspaper headlines, to blindness).

#### Hearing

Hearing was also determined using the classification (0 to 4) from the RAI assessment, where a higher score indicates more severe impairment.^28^ In this study, hearing was categorized into three different categories, where 0 indicates normal hearing (no problems in normal conversation), 1 suggests mild impairment (difficulties in some situations, e.g., when the other person speaks quietly), and scores 2 to 4 indicate moderate to severe impairment (problems hearing normal speech, to deafness).

#### Living arrangement

Living arrangement was determined using a single question, which included eight different options for living arrangements, such as living with a spouse, parents, children, or other relatives. In this study, the living arrangement was used as a binary variable: whether the person lived alone or with someone else.

#### Eye examination within the past year

Information on having an eye examination in the past year was determined using a single question: Have you had an eye examination within the past year?

### Statistical analysis

Descriptive analyses, such as frequencies, means, and standard deviations, were performed to get an overview of the sample. The assumption of multicollinearity was checked with inter-variable correlations ranging from −0.208 to 0.139. The dependent variable was vision examination in the past year, and the independent variables were age group, gender, living arrangement, cognition, physical function (ADL), vision, and hearing. The variables related to vision examination in the past year were studied using univariate and multivariate binary logistic regression analysis. The goodness of fit of the multivariate logistic regression model was assessed using the Hosmer–Lemeshow test (p>0.05). We analyzed data using IBM SPSS Statistics for Windows, Version 29.0 (IBM Corp., Armonk, NY). Statistical significance was defined at p<0.05.

## RESULTS

Most of the older HC clients (n = 19,150) were female (68%), nearly half were 85 years or older, and the majority (78%) lived alone (Table 1). One-fourth of HC clients had undergone an eye examination within the past year. Approximately 60% of HC clients had normal vision and hearing, while half of the clients had normal cognition and were physically independent in daily activities.

**TABLE 1.** Characteristics of study participants (n = 19,150)

	No eye examination within the past year (n = 14,114; 74%)	Eye examination within the past year (n = 5,036; 26%)	p-value*	Total (n = 19,150, 100%)
	n (%)	n (%)		
Gender			<0.001	
Male	4948 (35)	1595 (32)		6543 (34)
Female	9166 (65)	3441 (68)		12,607 (66)
Age			<0.001	
65–74	2292 (16)	702 (14)		2994 (16)
75–84	5080 (36)	1784 (35)		6864 (36)
≥85	6742 (48)	2550 (51)		9292 (49)
Living arrangement			0.02	
Living alone	10,994 (78)	4002 (80)		14,996 (78)
Living with someone	3120 (22)	1034 (21)		4154 (22)
Vision			<0.001	
Normal	9449 (67)	2734 (54)		12,183 (64)
Mild impairment	3742 (27)	1560 (31)		5302 (28)
Moderate or severe impairment	923 (7)	742 (15)		1665 (9)
Hearing			<0.001	
Normal	8732 (62)	2891 (57)		11,628 (61)
Mild impairment	3446 (24)	1390 (28)		4836 (25)
Moderate or severe impairment	1936 (14)	755 (15)		2691 (14)
Physical function (ADL)			<0.001	
Independent	7439 (53)	3124 (62)		10,563 (55)
Supervision or limited assistance	4455 (32)	1379 (27)		5832 (30)
Extensive assistance	2222 (16)	533 (11)		2755 (14)
Cognition			<0.001	
Normal	6408 (45)	2651 (53)		9059 (47)
Mild impairment	5020 (36)	1724 (34)		6744 (35)
Moderate or severe impairment	2686 (19)	661 (13)		3347 (18)

* Chi-square test. ADL = activities of daily living.

When comparing individuals who had undergone a vision examination within the past year to those who had not, statistically significant differences were found for all variables (Table 1). Females (68 vs. 65%), individuals aged 85 years and older (51 vs. 48%), individuals living alone (21 vs. 22%), and individuals with vision (46 vs. 33%) or hearing impairment (15 vs. 14%) had more often undergone an eye examination within the past year. Additionally, individuals with limited physical function in daily activities (38 vs. 47%), such as locomotion, and those with impaired cognition (47 vs. 55%) had less often undergone an eye examination within the past year.

Univariate and multivariate binary logistic regression models were used to analyze factors associated with having an eye examination within the past year among HC clients. The results of the univariate and multivariate binary logistic regression analysis are shown in Table 2. In the univariate models, older age, female gender, impaired vision, living alone, and impaired hearing were associated with higher odds of having an eye examination within the past year. In contrast, HC clients who had limited physical function or cognitive impairment had lower odds of having an eye examination within the past year. In the multivariate model, being female (odds ration [OR] 1.12; 95% confidence interval [CI] 1.04–1.20), having impaired vision, especially moderate or severe impairment (OR 3.01; 95% CI 2.70–3.36), and mild hearing impairment (OR 1.13; 95% CI 1.04–1.22) increased the odds of having had an eye examination within the last year. However, limited physical function, both needing supervision or limited assistance in ADL (OR 0.78; 95% CI 0.72–0.85) and needing extensive assistance (OR 0.60; 95% CI 0.53–0.67) and mild cognitive impairment (OR 0.86; 95% CI 0.80–0.92), as well as moderate or severe cognitive impairment (OR 0.66; 95% CI 0.59–0.74) were associated with lower odds of having an eye examination within the past year.

**TABLE 2.** Factors associated with having eye examination within the past year among older home care clients (n = 19,150). Analyzed with univariate and multivariate binary logistic regression models

	Univariate		Multivariate	
Variable	OR (95% CI)	p-value	OR (95% CI)	p-value
Age (ref. 65–74)				
75–84	1.15 (1.04–1.27)	**0.007**	1.11 (1.00–1.23)	0.054
≥85	1.24 (1.12–1.36)	**<0.001**	1.05 (0.95–1.17)	0.35
Gender (ref. male)	1.17 (1.09–1.25)	**<0.001**	1.12 (1.04–1.20)	**0.002**
Living arrangement (ref. living with someone)	1.10 (1.02–1.19)	**0.020**	0.98 (0.90–1.06)	0.56
Physical function (ref. independent)				
Supervision or limited assistance	0.74 (0.69–0.79)	**<0.001**	0.78 (0.72–0.85)	**<0.001**
Extensive assistance	0.57 (0.51–0.63)	**<0.001**	0.60 (0.53–0.67)	**<0.001**
Cognition (ref. no impairment)				
Mild impairment	0.83 (0.77–0.89)	**<0.001**	0.86 (0.80–0.92)	**<0.001**
Moderate or severe impairment	0.59 (0.54–0.65)	**<0.001**	0.66 (0.59–0.74)	**<0.001**
Vision (ref. no impairment)				
Mild impairment	1.44 (1.34–1.55)	**<0.001**	1.51 (1.40–1.63)	**<0.001**
Moderate or severe impairment	2.78 (2.50–3.09)	**<0.001**	3.01 (2.70–3.36)	**<0.001**
Hearing (ref. normal)				
Mild impairment	1.22 (1.13–1.31)	**<0.001**	1.13 (1.04–1.22)	**0.003**
Moderate or severe impairment	1.18 (1.07–1.29)	**<0.001**	1.04 (0.94–1.16)	0.42

Bold values indicate p-value <0.05. OR = odds ratio.

## DISCUSSION

The aim of this study was to explore the prevalence of older HC clients who have undergone an eye examination within the past year and the associated factors. The results showed that only 26% of HC clients have had an eye examination within the past year. This was lower compared with previous studies, in which the prevalence ranged from 37 to 39%. It seems that the international recommendation for eye examinations among older adults^4^ is not being met among older HC clients, raising concerns about the adequacy of their eye health and vision care services for this population. The findings also suggest that older HC clients with cognitive impairment or reduced physical function are less likely to have undergone an eye examination within the past year. A recent report highly recommends making screening and treatment for impaired vision accessible for all to reduce the risk of dementia in the life course.^5^

The findings of this study indicate that limited physical function hinders the ability of older HC clients to participate in eye examinations. These results align with a previous study that has demonstrated similar outcomes.^13^ Individuals with limited physical function often require more assistance and support for activities outside their home compared with their peers with normal physical function. While living with someone may provide additional help and support for older HC clients, the study did not find an association between living with someone and having eye examinations within the past year. This may be attributed to the fact that the live-in partner, often a spouse, may also have limitations of their own and may be unable to provide help with demanding tasks, such as transportation. As a practical suggestion, it is important to ensure that older HC clients are provided with adequate assistance and transfer services to overcome the challenges posed by limited physical function.

Since publicly funded transport services are continuously being reduced in Finland, one innovative solution for older HC clients with limited physical function to receive adequate eye care services could be mobile home-based eye care services. Currently, mobile home-based eye care services are scarcely available, even though Finland is a rapidly aging and sparsely populated country. By providing eye examinations in the home environment, eye care professionals could also pay attention to good contrast and adequate, high-quality lighting, issues that are essential in low vision rehabilitation.^32^ Well-designed lighting, such as minimizing shadows, is also important in creating dementia-friendly environments.^33^ Together with the best possible vision through up-to-date glasses or assistive optical devices, this could support safe and independent living at home.

Cognitive impairment was another factor associated with lower odds of having an eye examination within the past year, supporting findings from the previous study.^13^ The Lancet Standing Commission has recently published 14 modifiable risk factors for dementia, and untreated, later-life vision loss is one of them.^5^ Research has also revealed a bidirectional association between vision and cognition, indicating that individuals with impaired vision are more likely to experience cognitive impairment, and vice versa.^19^ Consequently, it becomes crucial to regularly assess vision in individuals with cognitive impairment, as addressing vision problems may potentially improve or slow down cognitive decline.^1,5^ Furthermore, this is crucial, especially in cases of existing hearing impairment.^5^ An encouraging finding of our study was that individuals with mild hearing impairment had more often undergone an eye examination than older HC clients with normal hearing. Additionally, it is important to prevent the combined impairment of vision, hearing, and/or cognition, as the existing study has demonstrated that combined impairment has more detrimental effects on overall functioning and care needs compared with a single impairment.^34^

Impaired vision was associated with having an eye examination within the past year, which is understandable. Nurses may have recommended an eye examination based on the results of the RAI assessment, as they play a central role in guiding older HC clients about eye health care services, given their close and regular contact with clients. While they can assist with booking an appointment for an eye examination, they are unable to accompany HC clients to external clinics. In such cases, support from a close one (relative or friend) is often necessary, which places those without such support in a particularly challenging and vulnerable position.

A previous study has shown that difficulties in prioritizing and incorporating routine eye examinations into existing health services are associated with their lower uptake.^35^ Although vision is regularly screened in HC using the RAI, there are no national guidelines or established care pathways for managing declined vision and visual impairment in HC. For example, there is a lack of information on whether HC clients are encouraged to undergo eye examinations even in the absence of symptoms. It is possible that HC nurses may not be aware that many eye diseases leading to permanent visual impairment, such as glaucoma, are initially asymptomatic.^36^ Therefore, targeted, specialized, and in-depth continuing education should be provided to HC nurses on vision and eye health, emphasizing the importance of regular eye examinations, delivered by vision and eye care specialists. In addition, it is essential to ensure that nurses feel qualified to conduct vision screenings, as suggested by previous studies,^37^ and to provide them with additional training when necessary. In Finland, vision health should be implemented into both nurses’ continuing education programs and their basic education, aligning with WHO guidelines that emphasize integration into primary health care.^38^ Continuing education for nurses could also benefit from the use of materials offered by established international programs and tele-education platforms (e.g., Cybersight).^39^

Implementing a standardized protocol for vision health in HC would also help ensure timely detection and management of vision impairment among this vulnerable population, thereby supporting safe living at home. There should also be a national discussion on the eye examinations at the recommended intervals for HC clients, on whether it should be annually, every 2 years, or whether an individually determined interval would be more appropriate, given that their vision is already screened using the RAI twice a year. Future research should focus on identifying the extent of unmet vision care needs among HC clients and developing strategies for early detection and treatment, considering the prevalence of eye care needs in this population.

One explanation for the low prevalence of participation in eye examinations could be Article 16 of the Health Care Professionals Decree 564/1994, which regulates optometrists’ practice. In the future, it will be interesting to observe how the repeal of this article affects the situation, as optometrists will be allowed to prescribe glasses for individuals who have undergone procedures such as cataract surgery. This change could potentially improve access to regular eye examinations among older adults, particularly those with low incomes. After the repeal of the article, it would also become easier to provide optometrist services at home. The private sector could complement the limited resources of public eye care services, but this would require stronger collaboration between the public and private sectors, as well as a clear division of responsibilities between optometrists and ophthalmologists. Furthermore, it is essential to ensure the sufficient competence of optometrists in their expanded role.

Eye examinations become increasingly important as individuals age due to the heightened risk of developing eye diseases.^3^ These examinations can identify potential eye diseases that may lead to permanent vision impairment and the burden of disability caused by it. Eye examinations not only help to prevent vision deterioration but also reduce the risk of other associated problems, such as limited physical functioning, cognitive impairment, falls, loneliness, and depression. Ultimately, eye examinations and adequate eye health care services may contribute positively to overall health and well-being, enabling older adults to live safely at home for longer. Preventing vision impairment with eye examinations also has economic implications that can be significant. However, in the future, the recommended interval for eye examinations among older HC clients should be more precisely defined.

### Strengths and limitations

This study had several strengths and limitations. A large dataset was one strength, as it may reduce the risk of potential inaccuracies. However, the data does not represent all older HC clients in Finland, as the use of the RAI assessment was voluntary in 2022. HC providers who chose to use RAI may have had certain characteristics that differed from those who did not. Thus, the findings may differ from those based on data from all HC units. One limitation was the lack of socio-economic information, which has been indicated to be associated with regular eye examinations in a previous study.^10^ In addition, the ADL variable used in this study does not comprehensively cover the aspects of physical functioning. Furthermore, a relationship between impaired cognition and ADL has been identified,^40^ which may influence the results, although multicollinearity of variables was low. Moreover, one limitation is the fact that in the RAI, vision is assessed based on near vision rather than distance vision, which may not provide a complete picture of an older adult’s overall visual ability. However, a previous study^41^ suggests that the correlation between near and distance visual acuity is strong among HC clients in Finland. Another possible limitation was the reliability of the data, as the subjective nature of RAI assessments and we could not verify how reliably the RAI assessments were conducted. However, RAIs are standardized and have been shown to have good validity and reliability.^42,43^ Nurses have also received training in conducting RAI assessments. One limitation is the cross-sectional design of the study, as it restricts making causal interpretations of the results. However, the results suggest that HC clients do not seek, may be reluctant, or may be unable to attend eye examinations at the recommended intervals due to various barriers. Further attention should be given to eye examinations at the recommended intervals for this population to prevent permanent vision impairment and manage current issues, along with their unwanted consequences.

## CONCLUSIONS

Many older HC clients have not participated in regular eye examinations within the past year. Eye examinations are important, as they are a way to maintain adequate vision, which is one key element to support healthy aging and potentially reduce the risk of dementia. Limited physical function and cognitive decline were found to be barriers to participating in eye examinations within the past year. As these challenges may hinder access to outside clinics, it is essential to ensure required support services, such as transportation and personal assistance, which can call for collaboration among HC staff, family members, and volunteers. Alternatively, eye care professionals could provide home-based eye care services for HC clients who have difficulty going to an outside clinic. One way to increase participation in eye examinations could be to increase the awareness and competence of HC professionals about eye care services and the importance of regular eye examinations.

The authors thank the Finnish Institute for Health and Welfare for providing the data used in this study.
